# Supplementary material for: Ocean freshening near the end of the Mesozoic
Source: Nat Commun. 2025 Aug 6;16:7238. doi: 10.1038/s41467-025-62189-9 (PMC12328591; doi:10.1038/s41467-025-62189-9)
Supplement: Supplementary file 1 — Supplementary Information [file 41467_2025_62189_MOESM1_ESM.pdf]

Supplementary Information
'Ocean freshening near the end of the Mesozoic'

Wiesława Radmacher *et al.*

*Corresponding author. Email: ndkrol@cyf-kr.edu.pl

This file includes Supplementary Figures 1 to 3 (SF1 to SF3), and Supplementary Note 1 (SN1) (all provided below). Supplementary Datasets 1 to 7 (SD1 to 7) are available as separate Excel files.

Supplementary Figure 1. Relative abundance of dinoflagellate cysts major groups throughout the Maastrichtian.
Supplementary Figure 2. Effects of Central American Seaway and Arctic Ocean bathymetry on global water salinity, temperature, and currents.
Supplementary Figure 3. Modelled precipitation-minus-evaporation (P-E) balance in the North Atlantic-Arctic region under different gateway configurations between the Arctic and proto-North Atlantic Ocean as well as varying CAS depths.
Supplementary Note 1. The bathymetry of the Central American Seaway in the Maastrichtian (ca. 70 Ma).
Supplementary Dataset 1. Palynological data from Sidi Ziane section, Algeria.
Supplementary Dataset 2. Age model, $\delta^{13}C$ and $\delta^{18}O$ stable isotopes, and $CaCO_3$ content for Sidi Ziane section, Algeria.
Supplementary Dataset 3. Planktic foraminiferal data from Sidi Ziane section, Algeria.
Supplementary Dataset 4. Palynological data from Re-2 core, Negev Region, Israel.
Supplementary Dataset 5. Palynological data from core 6711/4-U-1, Norwegian Sea.
Supplementary Dataset 6. Palynological data from core 6707/10-1, Norwegian Sea.
Supplementary Dataset 7. Dinoflagellate cysts relative abundance data from other localities.

Supplementary Figure 1 (SF1)

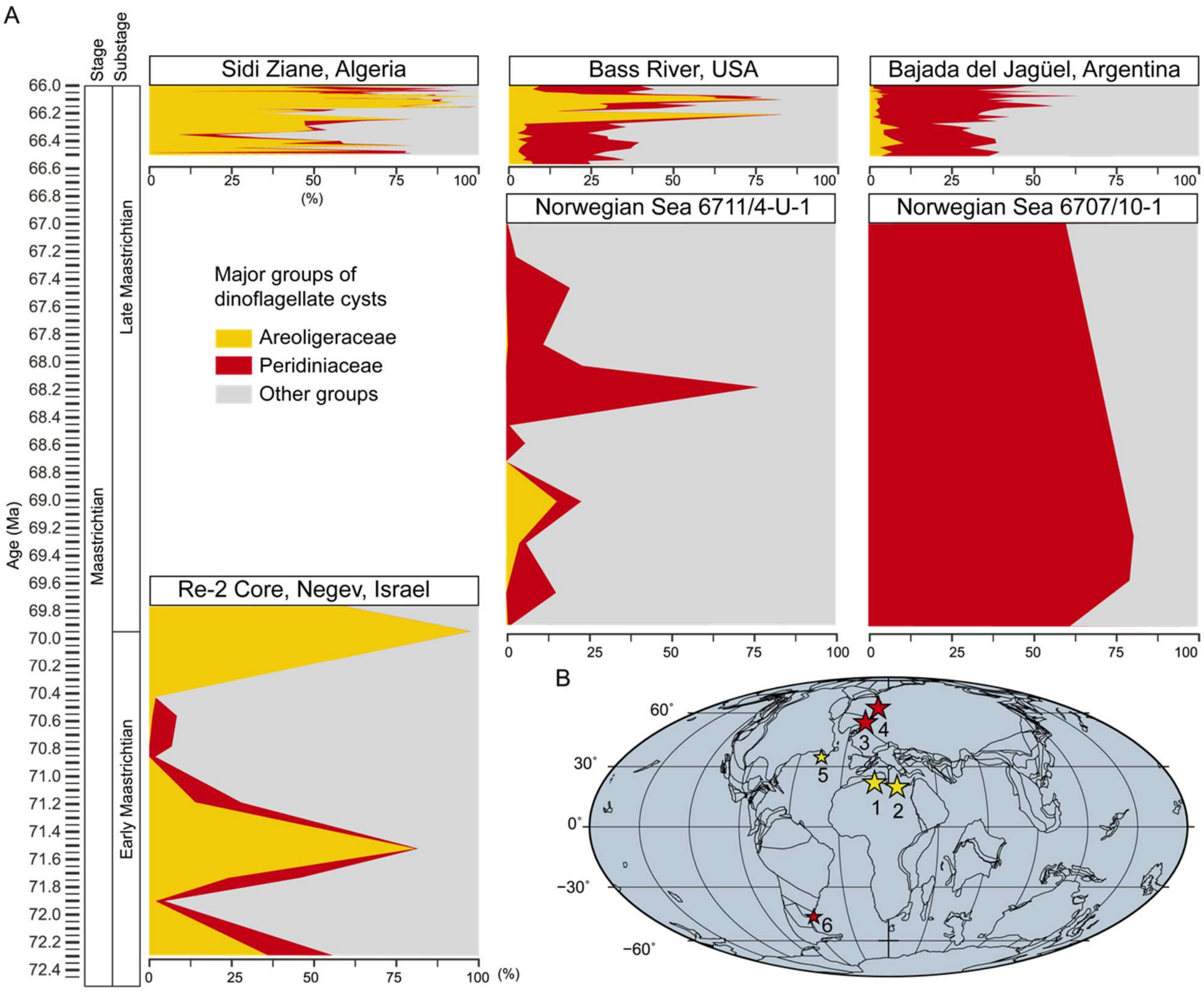

Supplementary Figure 1. Relative abundance of dinoflagellate cysts major groups throughout the Maastrichtian. 1. Sidi Ziane section, Algeria (this study); 2. Re-2 Core, Negev Region, Israel (this study); 3. Norwegian Sea, core 6707/10-1 (re-analyzed from Radmacher et al., 2015); 4. Norwegian Sea, core 6711/4-U-1 (re-analyzed from Radmacher et al., 2015); 5. Bass River, USA (Vellekoop et al., 2019); 6. Bajada del Jagüel, Argentina (Woelders et al., 2018). Red stars show high-latitude Maastrichtian sections dominated by Peridiniaceae. Yellow stars show low-latitude sections dominated by Areoligeraceae.

References are provided in the main text.

Supplementary Figure 2 (SF2)

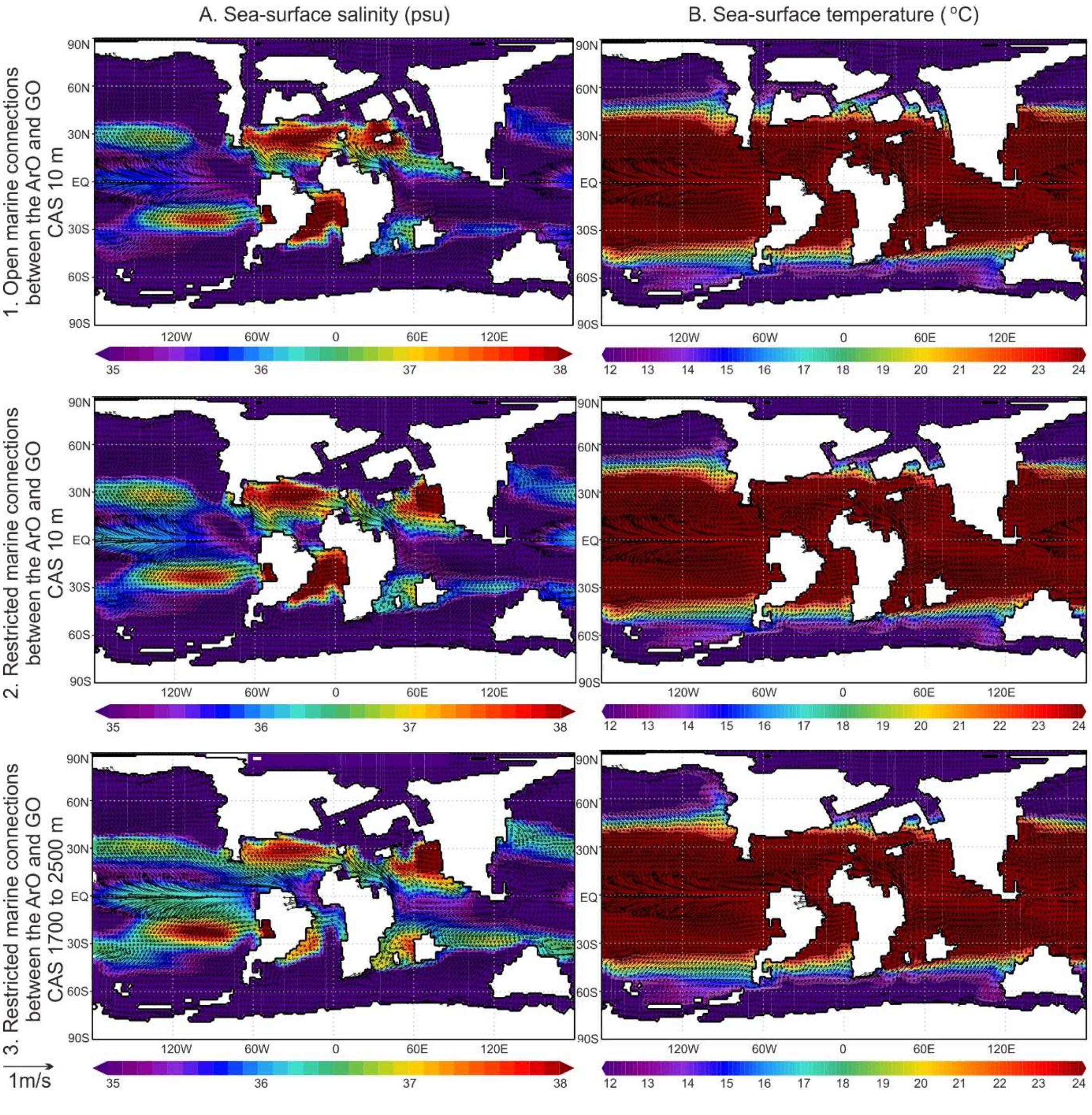

Supplementary Figure 2 I. Variations in global water surface salinity and temperature distributions due to water currents, driven by bathymetric changes in the Central American Seaway (CAS) and around the Arctic Ocean (ArO). The scale indicates the speed of water currents in meters per second (m/s). GO: Global Ocean.

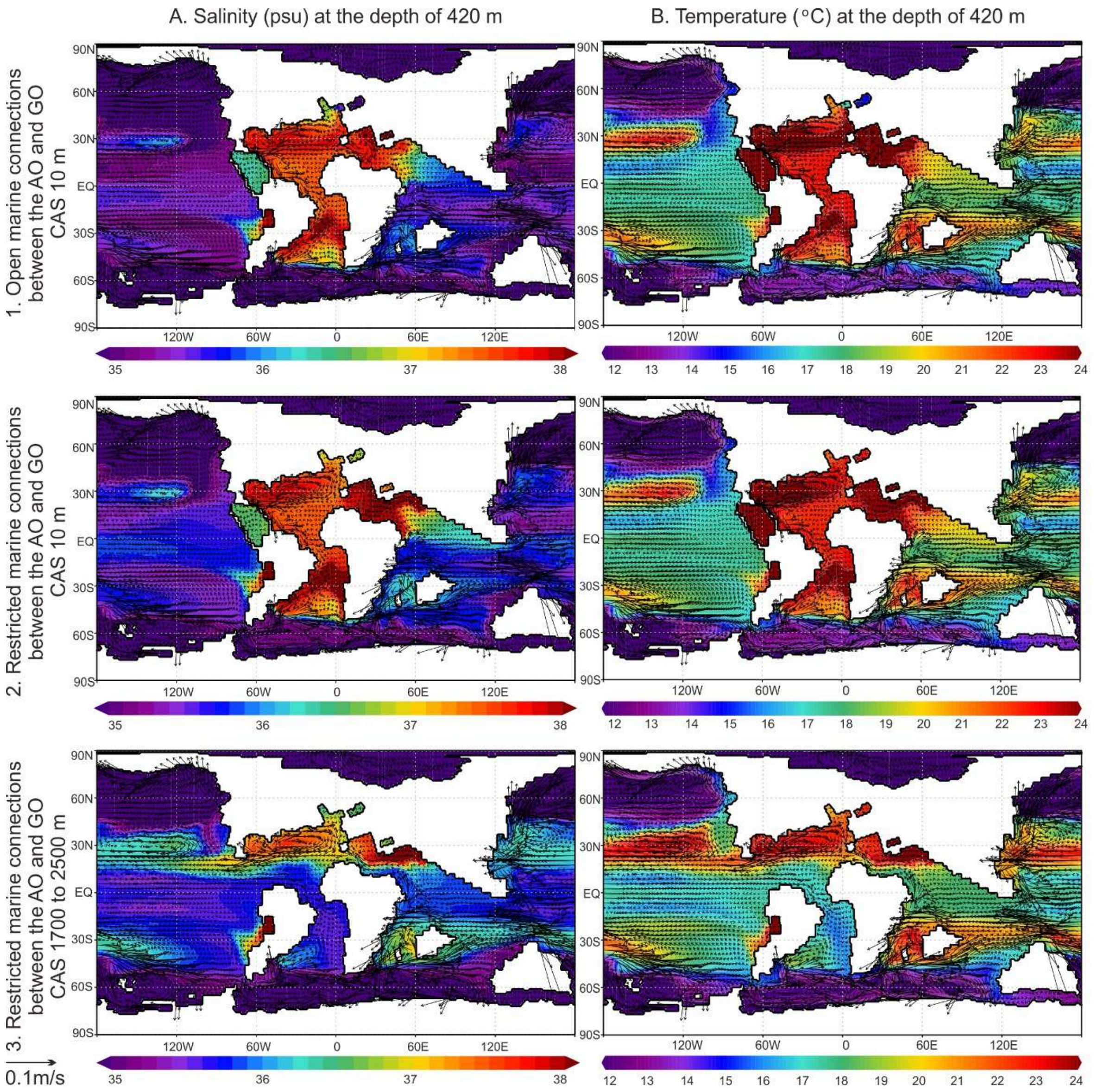

Supplementary Figure 2 II. Variations in global water salinity and temperature distributions at the depth of 420 meters, driven by bathymetric changes in the Central American Seaway (CAS) and restrictions around the Arctic Ocean (ArO). The scale indicates the speed of water currents in meters per second (m/s). GO: Global Ocean.

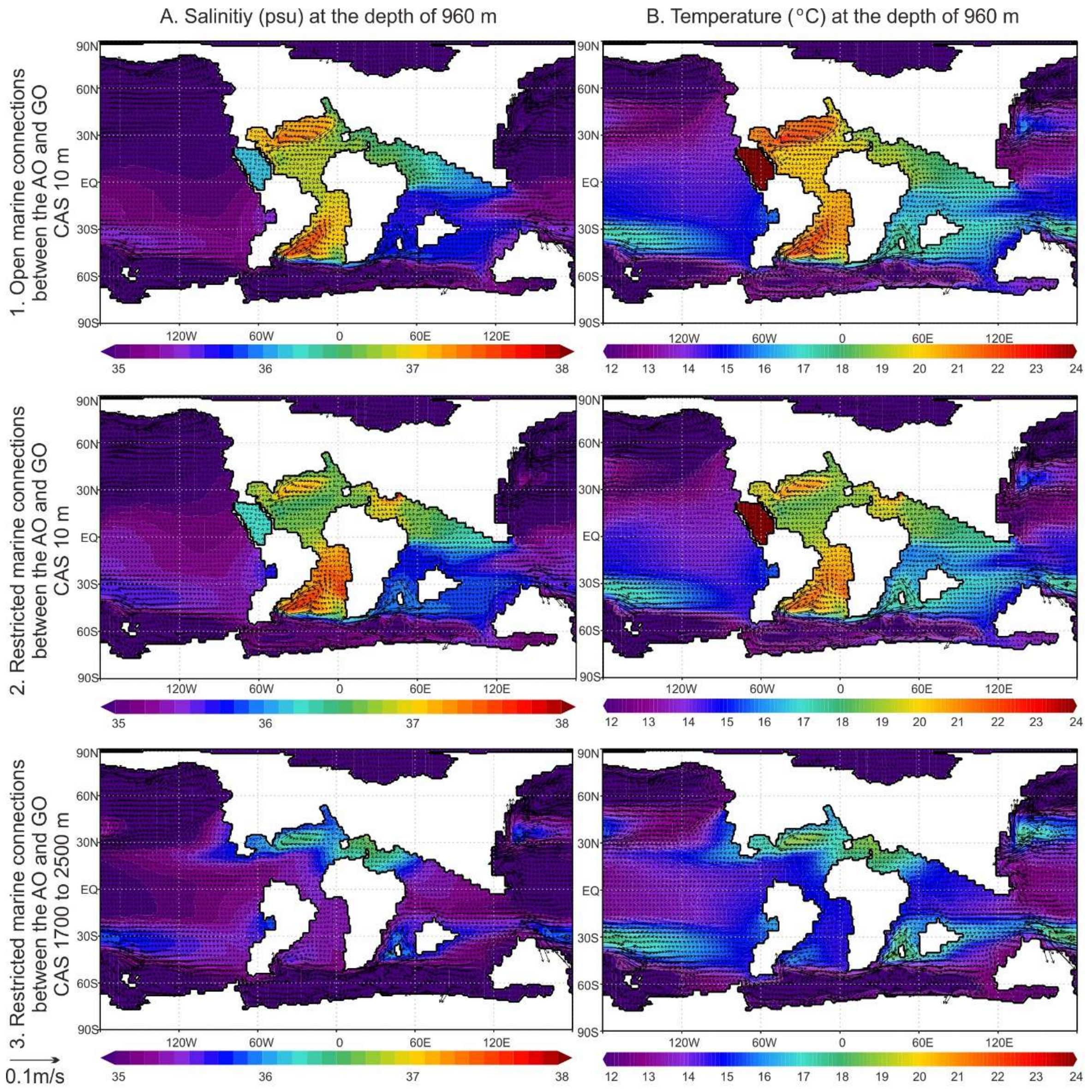

Supplementary Figure 2 III. Variations in global water salinity and temperature distributions at the depth of 960 meters, driven by bathymetric changes in the Central American Seaway (CAS) and restrictions around the Arctic Ocean (ArO). The scale indicates the speed of water currents in meters per second (m/s). GO: Global Ocean.

Supplementary Figure 3 (SF3)

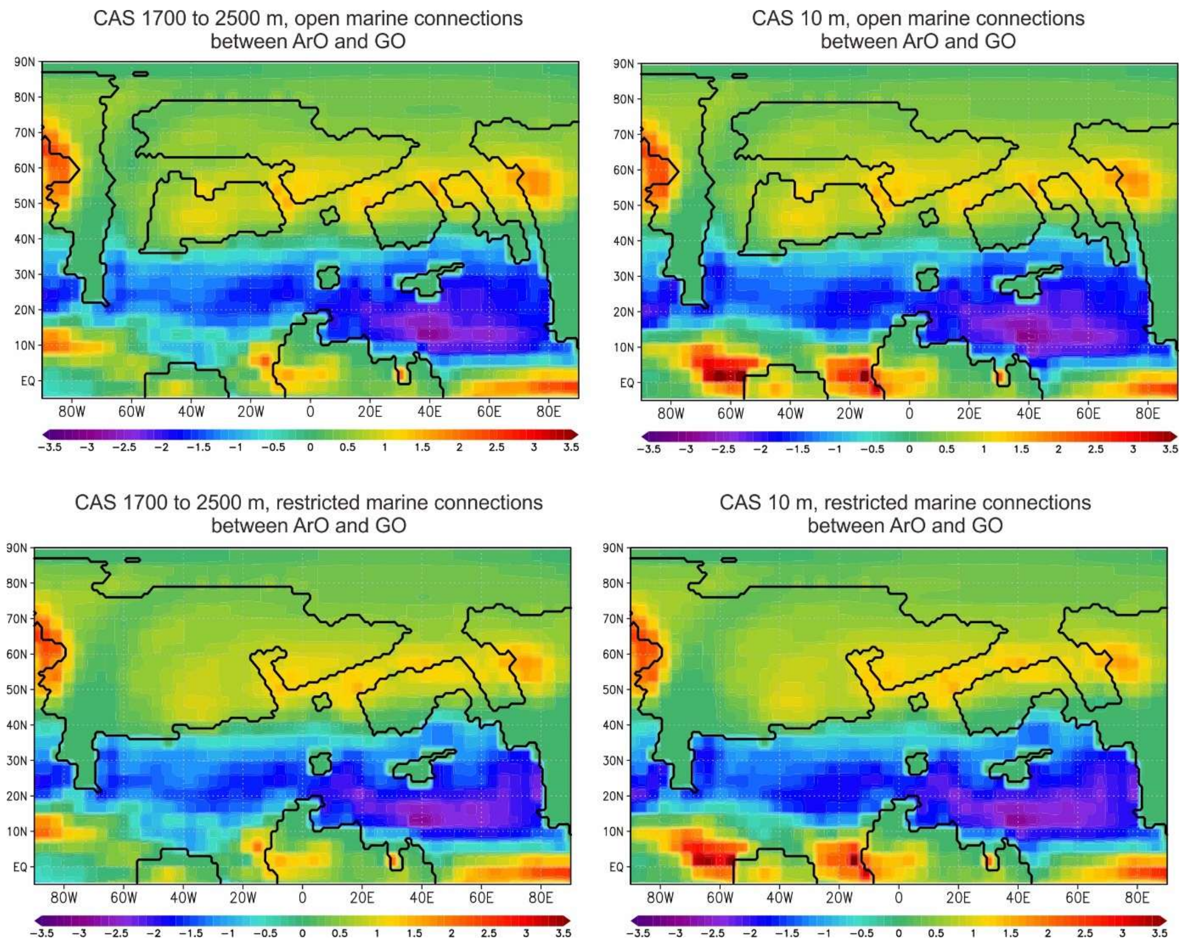

Supplementary Figure 3. Modelled precipitation-minus-evaporation (P-E) balance in the North Atlantic-Arctic region under different gateway configurations between the Arctic and proto-North Atlantic Ocean as well as varying CAS depths.

Supplementary Note 1 (SN1)

Depth characteristics of the Central American Seaway

The bathymetry of the Central American Seaway (CAS) in the Maastrichtian (~70 Ma) is constrained by (1) regional geological observations that support the occurrence of an island arc system between the North and South American continents (referred to as "CAS island arc" thereafter), and (2) a compilation of regional bathymetry along several modern island arcs that provide an independent conservative estimate of the paleodepth of the CAS island arc.

The latest Cretaceous CAS island arc corresponds to the oceanic segment of modern southern Central America that mostly includes Costa Rica and Panama. It is currently located between Nicaragua and Colombia that represent the southern and northern edges of North and South American continents, respectively (Kennan and Pindell, 2009). The northern termination of the CAS island arc was juxtaposed to the Chortis Block along the Mesquito Composite Terrane in the latest Cretaceous (Baumgartner et al., 2008; Andjić et al., 2019), with shallow-marine conditions documented in the nearby continental domain of the Chortis Block (e.g., Rogers et al., 2007). The CAS volcanic arc (and the Caribbean oceanic plateau that forms its basement, see below) was colliding with Ecuador-Colombia in the latest Cretaceous (Vallejo et al., 2009; Pardo-Trujillo et al. 2000), with shallow-marine conditions documented in the nearby continental area of South America (e.g., Calderon-Díaz et al., 2024). Therefore, a paleodepth of 50 m is consistent with the geological record documented in continental areas contiguous to the CAS island arc in the Maastrichtian.

Two lines of geological evidence support shallow marine environments in the CAS island arc in the Maastrichtian. First, it is well documented that shallow marine conditions have existed in several areas between Costa Rica and Panama since at least the Eocene (Alfaro et al., 2023; Barat et al., 2014; Buchs et al., 2011; Montes et al., 2012a), suggesting that the island arc was already forming a significant inter-oceanic barrier by this time. In addition, explosive shallow-marine to subaerial supra-subduction volcanism is documented by tuffaceous/volcaniclastic components found in Campanian-Maastrichtian hemipelagic limestones (Buchs et al., 2010, Corral et al., 2013) or latest Cretaceous-earliest Paleogene submarine volcanic units (Di Marco et al., 1995; Wörner et al., 2005) associated with the early volcanic arc. These direct geological observations clearly support the occurrence of an island arc in the latest Cretaceous. The second line of geological evidence is that the CAS island arc developed on top of the Caribbean oceanic plateau, as evidenced by volcanic arc dykes cross-cutting oceanic plateau sequences between south Costa Rica and central Panama (Buchs et al., 2010; Montes et al., 2012b). Formation of the Caribbean oceanic plateau ca. 90 Ma ago was associated with the formation of volcanic islands (Buchs et al., 2018, and references therein), therefore suggesting that it still formed an oceanic promontory that could have facilitated shallow marine to subaerial environments along the CAS island arc in the latest Cretaceous.

Bathymetric data were measured at several modern island arcs using GeoMapApp (Ryan et al., 2009, data retrieved in 2019 using the application available at www.geomapapp.org) to further estimate realistic paleodepth at the CAS island arc in the Maastrichtian. As illustrated in the table and figures below, an average island arc paleodepth at the scale considered in our modelling can range between approximately 1800 and 200 meters below sea level. These paleodepths should be regarded as maximum possible estimates because modern island arcs have developed on ocean floor formed at mid ocean ridges or during back-arc opening, but the CAS island arc developed on top of an oceanic plateau promontory that most likely facilitated volcanic shallowing and

emergence. Therefore, we conclude based on regional geological constraints and the typical bathymetry of island arcs that the most likely average paleodepth of the CAS island arc in the Maastrichtian was between 500 and 50 m.

Summary of average island arc depths (details given in the figures below):

	Tonga	Izu-Bonin	Mariana	Kuril	Aleutian	Antilles
**Arc segment (km)**	1058	1182	1350	1144	2166	823
**Segment width (km)**	70	70	70	100	70	130
**Average depth (m)**	-1264	-1778	-1817	-410	-518	-195

Volcanic arc segments used in the table above (from GeoMapApp, Ryan et al., 2009):

Tonga:

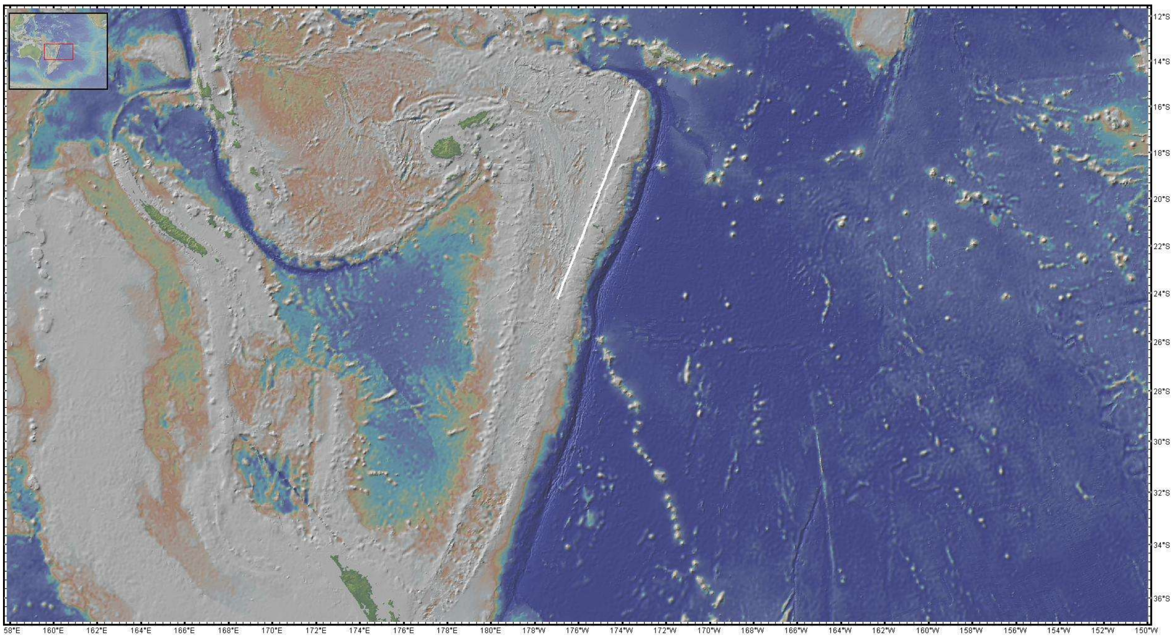

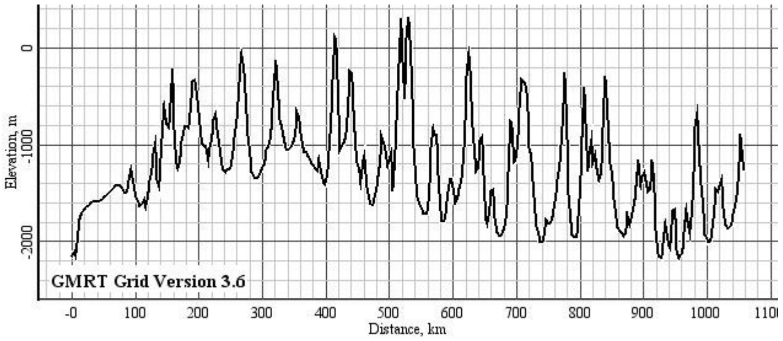

Izu-Bonin:

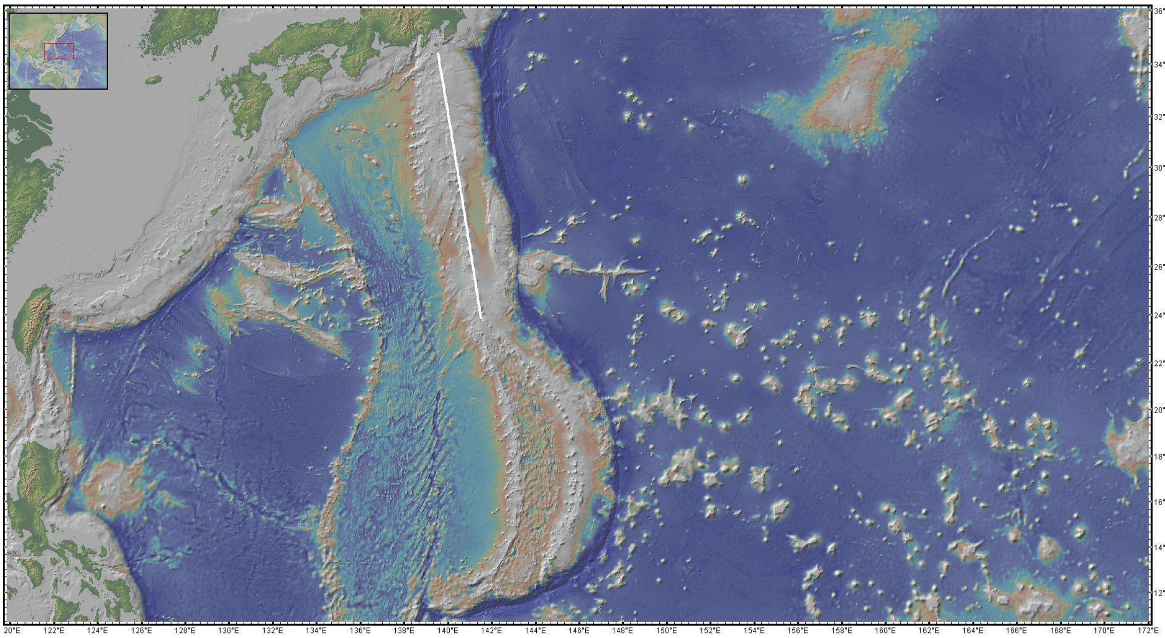

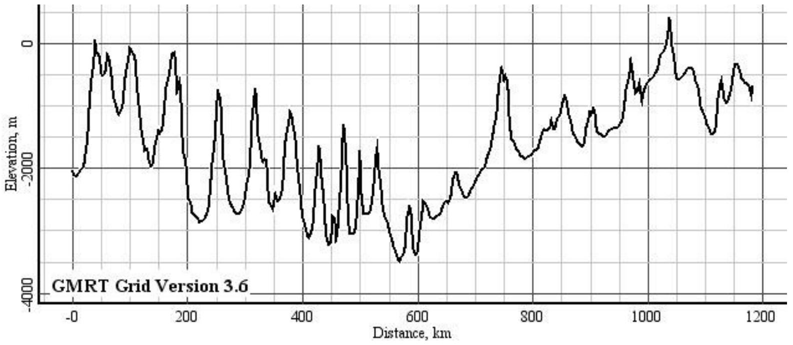
Elevation, m
0
-2000
-4000
GMRT Grid Version 3.6
-0
200
400
600
800
1000
1200
Distance, km

Kuril:

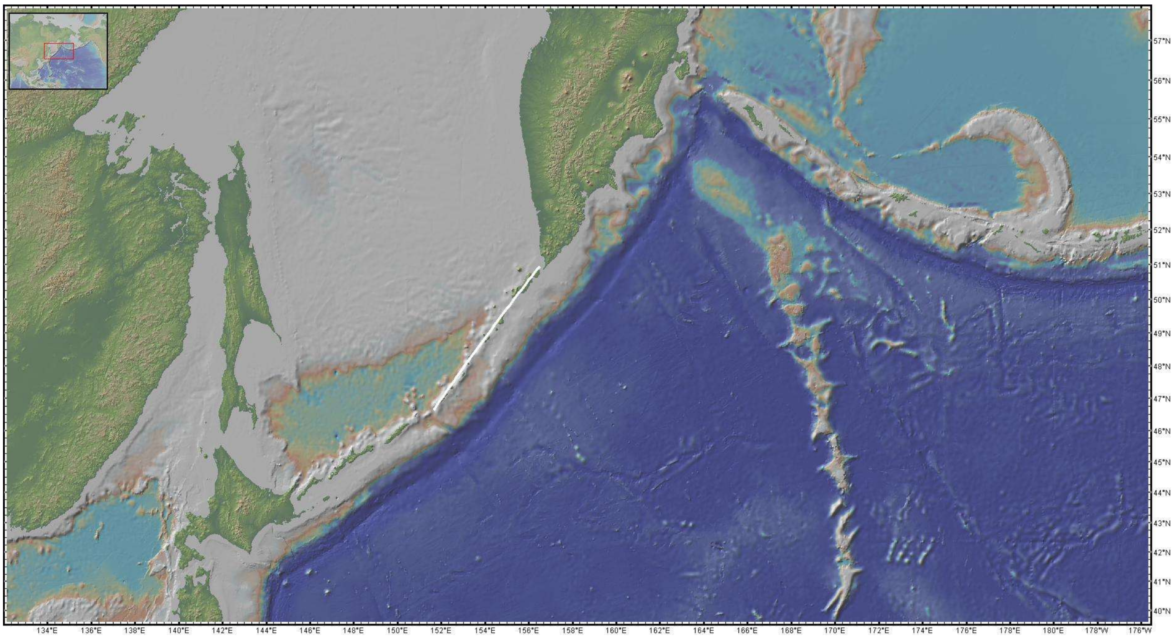

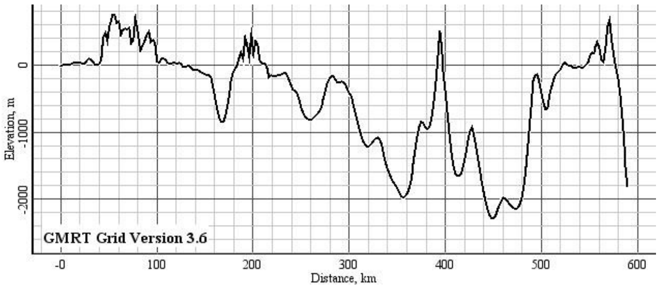

GMRT Grid Version 3.6
Elevation, m
0
-1000
-2000
-0
100
200
300
400
500
600
Distance, km

Aleutian:

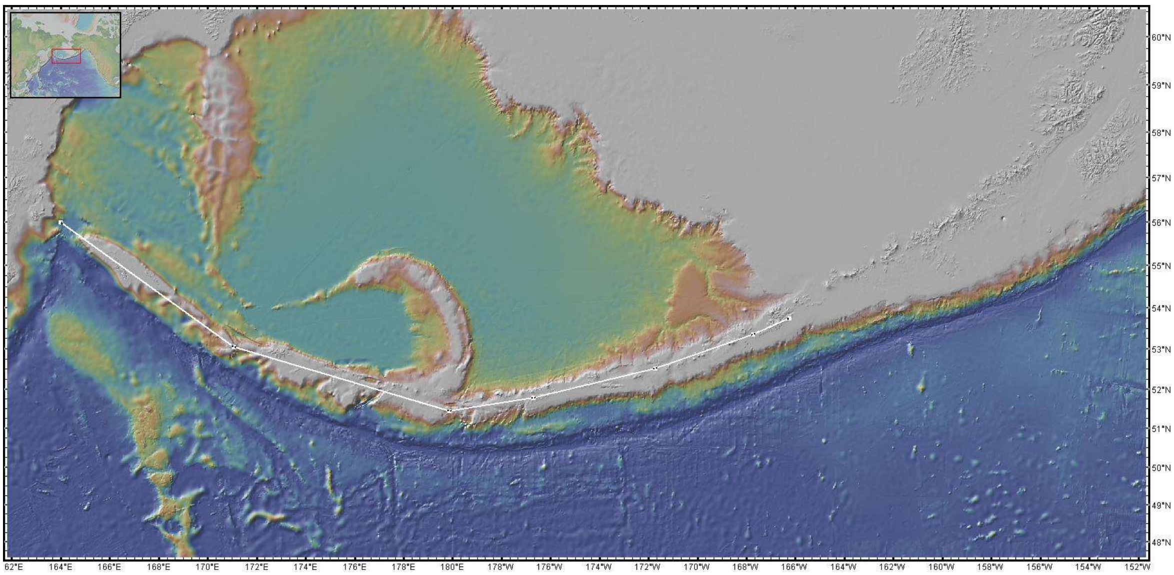
60°N
59°N
58°N
57°N
56°N
55°N
54°N
53°N
52°N
51°N
50°N
49°N
48°N
164°E 166°E 168°E 170°E 172°E 174°E 176°E 178°E 180°E 178°W 176°W 174°W 172°W 170°W 168°W 166°W 164°W 162°W 160°W 158°W 156°W 154°W 152°W

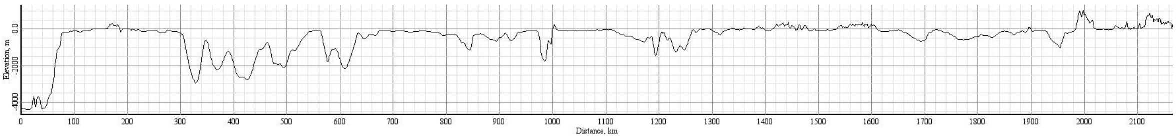
Elevation, m
0.0
-2000
-4000
0 100 200 300 400 500 600 700 800 900 1000 1100 1200 1300 1400 1500 1600 1700 1800 1900 2000 2100
Distance, km

Antilles:

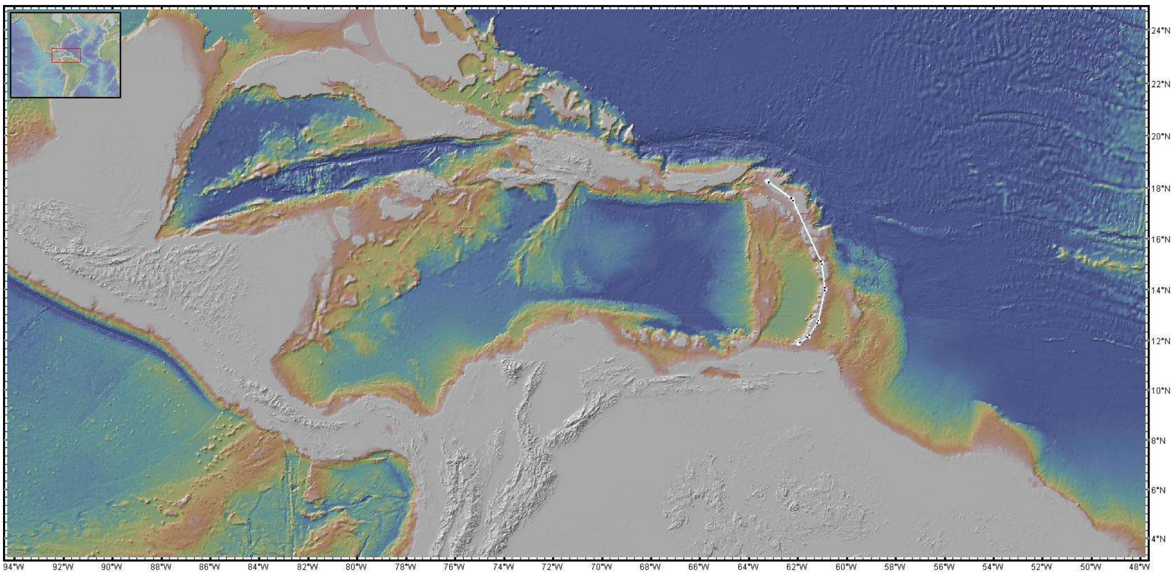
24°N
22°N
20°N
18°N
16°N
14°N
12°N
10°N
8°N
6°N
4°N
94°W
92°W
90°W
88°W
86°W
84°W
82°W
80°W
78°W
76°W
74°W
72°W
70°W
68°W
66°W
64°W
62°W
60°W
58°W
56°W
54°W
52°W
50°W
48°W

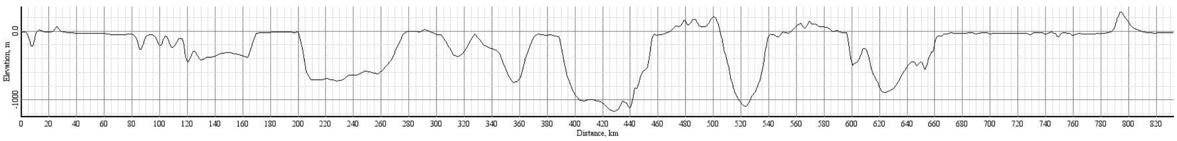
Elevation, m
0.0
-1000
Distance, km

Supplementary References

1. Alfaro, A., Denyer, P., Calvo, C., Bolz, A., Aguilar, T., Vargas, C., Chesnel, V., Rodríguez, E., 2023. Tectonostratigraphic model of the Fila Costeña in southern Central America based on new biostratigraphic data: implications of a two-thrust-fault hypothesis. Journal of South American Earth Sciences 128, 104465. https://doi.org/10.1016/j.jsames.2023.104465
2. Andjić, G., Baumgartner, P.O., Baumgartner-Mora, C., 2019. Collision of the Caribbean Large Igneous Province with the Americas: Earliest evidence from the forearc of Costa Rica. GSA Bulletin. https://doi.org/10.1130/B35037.1
3. Barat, F., Mercier de Lépinay, B., Sosson, M., Müller, C., Baumgartner, P.O., Baumgartner-Mora, C., 2014. Transition from the Farallon Plate subduction to the collision between South and Central America: Geological evolution of the Panama Isthmus. Tectonophysics 622, 145–167. http://dx.doi.org/10.1016/j.tecto.2014.03.008
4. Baumgartner, P.O., Flores, K., Bandini, A., Girault, F., Cruz, D., 2008. Upper Triassic to Cretaceous Radiolaria from Nicaragua and Northern Costa Rica - The Mesquito Composite Oceanic Terrane. Ofioliti 33, 1–19.
5. Buchs, D.M., Arculus, R.J., Baumgartner, P.O., Baumgartner-Mora, C., Ulianov, A., 2010. Late Cretaceous arc development on the SW margin of the Caribbean Plate: Insights from the Golfito, Costa Rica, and Azuero, Panama, complexes. Geochemistry Geophysics Geosystems 11, Q07S24. https://doi.org/10.1029/2009gc002901
6. Buchs, D.M., Baumgartner, P.O., Baumgartner-Mora, C., Flores, K., Bandini, A.N., 2011. Upper Cretaceous to Miocene tectonostratigraphy of the Azuero area (Panama) and the discontinuous accretion and subduction erosion along the Middle American margin. Tectonophysics 512, 31–46.
7. Buchs, D.M., Kerr, A.C., Brims, J.C., Zapata-Villada, J.P., Correa-Restrepo, T., Rodríguez, G., 2018. Evidence for subaerial development of the Caribbean oceanic plateau in the Late Cretaceous and palaeo-environmental implications. Earth and Planetary Science Letters 499, 62–73. https://doi.org/10.1016/j.epsl.2018.07.020
8. Calderon-Diaz, L., Zapata, S., Cardona, A., Parra, M., Sobel, E.R., Patiño, A.M., Valencia, V., Jaramillo-Rios, J.S., Glodny, J., 2024. Cretaceous extensional and contractional stages in the Colombian Andes unraveled by a source-to-sink geochronological and thermochronological study in the Upper Magdalena Basin. Tectonophysics 878, 230303. https://doi.org/10.1016/j.tecto.2024.230303
9. Corral, I., Gómez-Gras, D., Griera, A., Corbella, M., Cardellach, E., 2013. Sedimentation and volcanism in the Panamanian Cretaceous intra-oceanic arc and fore-arc: New insights from the Azuero peninsula (SW Panama). Bulletin De La Societe Geologique De France 184, 35–45. https://doi.org/10.2113/gssgfbull.184.1-2.35
10. Di Marco, G., Baumgartner, P.O., Channell, J.E.T., 1995. Late Cretaceous-early Tertiary paleomagnetic data and a revised tectonostratigraphic subdivision of Costa Rica and western Panama.; Geologic and tectonic development of the Caribbean Plate boundary in southern Central America, in: Mann, P. (Ed.), Geologic and Tectonic Development of the Caribbean Plate Boundary in Southern Central America, Geological Society of America Special Paper. pp. 1–27.
11. Kennan, L., Pindell, J., 2009. Dextral shear, terrane accretion and basin formation in the Northern Andes: explained only by interaction with a Pacific-derived Caribbean Plate, in: James, K.H., Lorente, M.A., Pindell, J. (Eds.), The Origin and Evolution of the Caribbean Plate, Geological Society of London (Special Publication). The Geological Society of London, pp. 487–531.
12. Montes, C., Cardona, A., McFadden, R., Morón, S.E., Silva, C.A., Restrepo-Moreno, S., Ramírez, D.A., Hoyos, N., Wilson, J., Farris, D., Bayona, G.A., Jaramillo, C.A., Valencia, V., Bryan, J., Flores, J.A., 2012a.

Evidence for middle Eocene and younger land emergence in central Panama: Implications for Isthmus closure. Geological Society of America Bulletin. https://doi.org/10.1130/b30528.1

13. Montes, C., Bayona, G., Cardona, A., Buchs, D.M., Silva, C.A., Morón, S., Hoyos, N., Ramírez, D.A., Jaramillo, C.A., Valencia, V., 2012b. Arc-continent collision and orocline formation: Closing of the Central American seaway. Journal of Geophysical Research 117, B04105.
14. Pardo-Trujillo, A., Cardona, A., Giraldo, A.S., León, S., Vallejo, D.F., Trejos-Tamayo, R., Plata, A., Ceballos, J., Echeverri, S., Barbosa-Espitia, A., Slattery, J., Salazar-Ríos, A., Botello, G.E., Celis, S.A., Osorio-Granada, E., Giraldo-Villegas, C.A., 2020. Sedimentary record of the Cretaceous–Paleocene arc–continent collision in the northwestern Colombian Andes: Insights from stratigraphic and provenance constraints. Sedimentary Geology 401, 105627. https://doi.org/10.1016/j.sedgeo.2020.105627
15. Rogers, R.D., Mann, P., Scott, R.W., Patino, L., 2007. Cretaceous intra-arc rifting, sedimentation, and basin inversion in east-central Honduras. Geological Society of America Special Papers 428, 89–128. https://doi.org/10.1130/2007.2428(05)
16. Ryan, W.B.F., Carbotte, S.M., Coplan, J.O., O'Hara, S., Melkonian, A., Arko, R., Weissel, R.A., Ferrini, V., Goodwillie, A., Nitsche, F., Bonczkowski, J., Zemsky, R., 2009. Global Multi-Resolution Topography synthesis. Geochemistry, Geophysics, Geosystems 10. https://doi.org/10.1029/2008GC002332
17. Vallejo, C., Winkler, W., Spikings, R.A., Luzieux, L., Heller, F., Bussy, F., 2009. Mode and timing of terrane accretion in the forearc of the Andes in Ecuador, in: Kay, S.M., Ramos, V.A., Dickinson, W.R. (Eds.), Backbone of the Americas: Shallow Subduction, Plateau Uplift, and Ridge and Terrane Collision. pp. 197–216.
18. Wörner, G., Harmon, R.S., Hartmann, G., Simon, K., 2005. Igneous Geology and Geochemistry of the Upper Río Chagres Basin, in: Singh, V.P., Harmon, R.S. (Eds.), The Río Chagres, Panama: A Multidisciplinary Profile of a Tropical Water
