# Supplementary material for: Ocean freshening near the end of the Mesozoic
Source: Nat Commun. 2025 Aug 6;16:7238. doi: 10.1038/s41467-025-62189-9 (PMC12328591; doi:10.1038/s41467-025-62189-9)
Supplement: Supplementary file 2 — Description of Additional Supplementary Files [file 41467_2025_62189_MOESM2_ESM.pdf]

Supplementary Figure 1 (SF1)

Relative abundance of dinoflagellate cysts major groups throughout the Maastrichtian. 1. Sidi Ziane section, Algeria (this study); 2. Re-2 Core, Negev Region, Israel (this study); 3. Norwegian Sea, core 6707/10-1 (re-analyzed from Radmacher et al., 2015); 4. Norwegian Sea, core 6711/4-U-1 (re-analyzed from Radmacher et al., 2015); 5. Bass River, USA (Vellekoop et al., 2019); 6. Bajada del Jagüel, Argentina (Woelders et al., 2018). Red stars show high-latitude Maastrichtian sections dominated by Peridiniaceae. Yellow stars show low-latitude sections dominated by Areoligeraceae.

Supplementary Figure 2 (SF2)

Effects of Central American Seaway (CAS) and Arctic Ocean (ArO) bathymetry on global water salinity, temperature, and currents. S2 I: Variations in global water surface salinity and temperature distributions due to water currents, driven by bathymetric changes in the CAS and around the ArO. S2 II: Variations in global water salinity and temperature distributions at the depth of 420 meters, driven by bathymetric changes in the CAS and restrictions around the ArO. S2 III: Variations in global water salinity and temperature distributions at the depth of 960 meters, driven by bathymetric changes in the CAS and restrictions around the ArO. The scale indicates the speed of water currents in meters per second (m/s). GO: Global Ocean.

Supplementary Figure 3 (SF3)

Modelled precipitation-minus-evaporation (P-E) balance in the North Atlantic-Arctic region under different gateway configurations between the Arctic and proto-North Atlantic Ocean as well as varying CAS depths.

Supplementary Note 1 (SN1)

The bathymetry of the Central American Seaway (CAS) in the Maastrichtian (ca. 70 Ma).

Supplementary Dataset 1 (SD1)

Palynological data from Sidi Ziane section, Algeria.

Supplementary Dataset 2 (SD2)

Age model, $\delta^{13}C$ and $\delta^{18}O$ stable isotopes, and $CaCO_3$ content for Sidi Ziane section, Algeria.

Supplementary Dataset 3 (SD3)

Planktic foraminiferal data from Sidi Ziane section, Algeria.

Supplementary Dataset 4 (SD4)

Palynological data from Re-2 core, Negev Region, Israel.

Supplementary Dataset 5 (SD5)

Palynological data from core 6711/4-U-1, Norwegian Sea.

Supplementary Dataset 6 (SD6)

Palynological data from core 6707/10-1, Norwegian Sea.

Supplementary Dataset 7 (SD7)

Dinoflagellate cysts relative abundance data from other localities.
